# Supplementary material for: Recessive TMOD1 mutation causes childhood cardiomyopathy
Source: Commun Biol. 2024 Jan 2;7:7. doi: 10.1038/s42003-023-05670-9 (PMC10761686; doi:10.1038/s42003-023-05670-9)
Supplement: Supplementary file 2 — Supplementary Information [file 42003_2023_5670_MOESM2_ESM.pdf]

Supplementary Information

Recessive *TMOD1* mutation causes childhood cardiomyopathy

Catalina Vasilescu[#], Mert Colpan[#], Tiina H. Ojala, Tuula Manninen, Aino Mutka, Kaisa Ylänen, Otto Rahkonen, Tuija Poutanen, Laura Martelius, Reena Kumari, Helena Hinterding, Virginia Brilhante, Simo Ojanen, Pekka Lappalainen, Juha Koskenvuo, Christopher J. Carroll, Velia M. Fowler, Carol C. Gregorio[*], Anu Suomalainen[*]

Supplementary Table 1. Dissociation constants (K_d, µM or nM) for inhibition of pointed end polymerization or depolymerization of actin filaments in the absence (-) or presence (+) of TPM1 by TMOD1^{wt} or TMOD1^{R189W} when filament barbed ends are capped by gelsolin at a gelsolin-to-actin molar ratio of 1:10. Data are presented as mean ± standard deviation (n=3 independent experiments). Statistically significant differences are highlighted in bold (Student's t-test, **p<0.05, *p<0.01*).

	TPM1	TMOD1^{wt}	TMOD1^{R189W}	*p*-value	Fold difference	Source Figure
Pyrene-actin polymerization	-	0.49 ± 0.09 µM	**1.06 ± 0.10 µM****	0.0019	~2.2	Fig. 4b
	+	6.63 ± 0.07 nM	**9.88 ± 0.72 nM****	0.0014	~1.5	Fig. 4d
Pyrene-actin depolymerization	-	0.24 ± 0.06 µM	**1.13 ± 0.19 µM****	0.0016	~4.7	Fig. 4f
	+	25.3 ± 5.8 nM	**79.4 ± 25.6 nM***	0.0233	~3.2	Fig. 4h

Supplementary Table 2. Dissociation constants (K_d, μM or nM) for inhibition of pointed end polymerization or depolymerization of actin filaments in the absence (-) of TPM1 by $TMOD1^{wt}$ or $TMOD1^{R189W}$ when filament barbed ends are capped by gelsolin at a gelsolin-to-actin molar ratio of 1:100. Data are presented as mean ± ± standard deviation (n=3 independent experiments). Statistically significant differences are highlighted in bold (Student's t-test, *$p<0.05$, *$p<0.01$*).

	TPM1	$TMOD1^{wt}$	$TMOD1^{R189W}$	*p*-value	Fold difference	Source Figure
Pyrene-actin polymerization	-	1.03 ± 0.31 μM	**1.99 ± 0.18 μM****	0.0099	~2.0	Supp. Fig. 6b
Pyrene-actin depolymerization	-	0.73 ± 0.09 μM	**2.00 ± 0.58 μM***	0.0192	~2.8	Supp. Fig. 6d

Supplementary Table 3. The concentration required for 50% inhibition of Lat A-induced actin pointed end depolymerization in the presence (+) of TPM1 by $TMOD1^{wt}$ or $TMOD1^{R189W}$ (IC_{50}) when filament barbed ends are capped by gelsolin. The IC_{50} values were calculated from actin band densities of pellet and supernatant samples following sedimentation of filaments. Data are presented as mean ± ± standard deviation (n=3 or 4 independent experiments). Statistically significant differences are highlighted in bold (Student's t-test, *$p<0.05$).

	TPM1	$TMOD1^{wt}$	$TMOD1^{R189W}$	*p*-value	Fold difference	Source Figure
Pellet	+	9.8 ± 2.1 nM	**23.0 ± 7.6 nM***	0.0160	~2.4	Supp. Fig. 7b
Supernatant	+	11.1 ± 3.2 nM	**35.1 ± 14.2 nM***	0.0455	~3.2	Supp. Fig. 7d

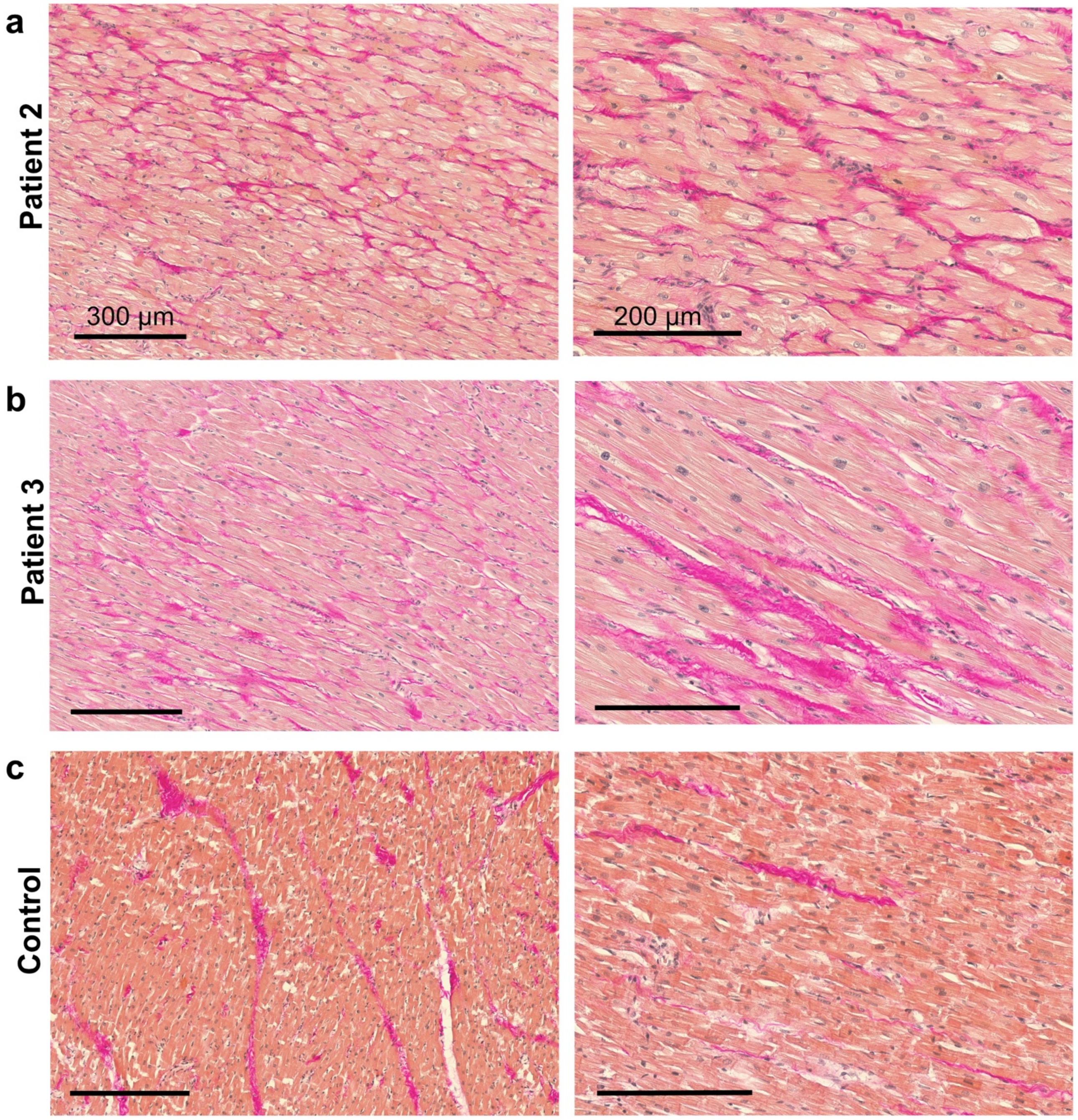

Supplementary Figure 1. Herovici staining of cardiac tissue at 100x (left) and 200x (right) magnification. **a** Patient 2, operated on at the age 13 years. **b** Patient 3, operated on at the age of 12.5 years. **c** Control heart tissue, autopsy at the age of 10 years. In patients, areas of perimyocytic fibrosis as well as wavy-arranged myocardium are observed. There is a variation in the size and shape of nuclei, enlarged in the patients, with focally perinuclear clearing. Perimyocytic fibrosis and myodegeneration were more prominent in Patient 2 than in Patient 3.

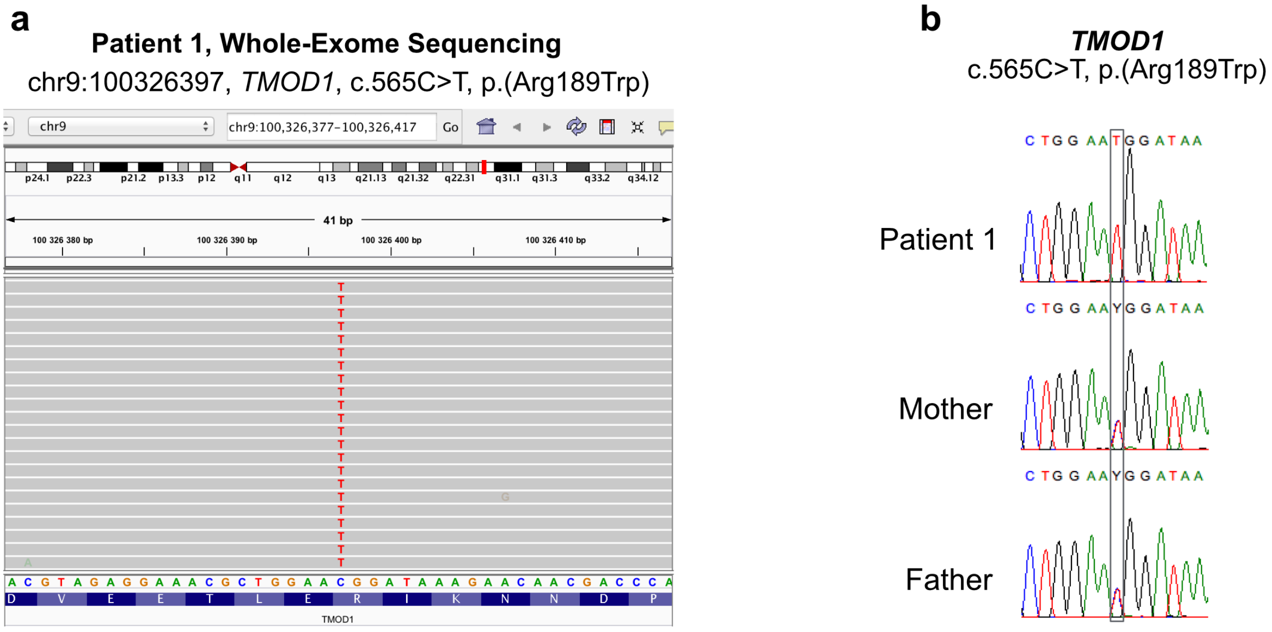

Supplementary Figure 2. Homozygous variant c.565C>T, p.Arg198Trp in *TMOD1*, Family 1. a Whole-exome data of Patient 1 showing the variant position, visualized with Integrated Genome Viewer. **b** Sanger chromatograms of the variant segregation in Family 1. The peaks for A, T, G and C are shown in green, red, black and blue, respectively.

Family 2, Whole-Exome Sequencing

chr9:100326397, *TMOD1*, c.565C>T, p.Arg189Trp

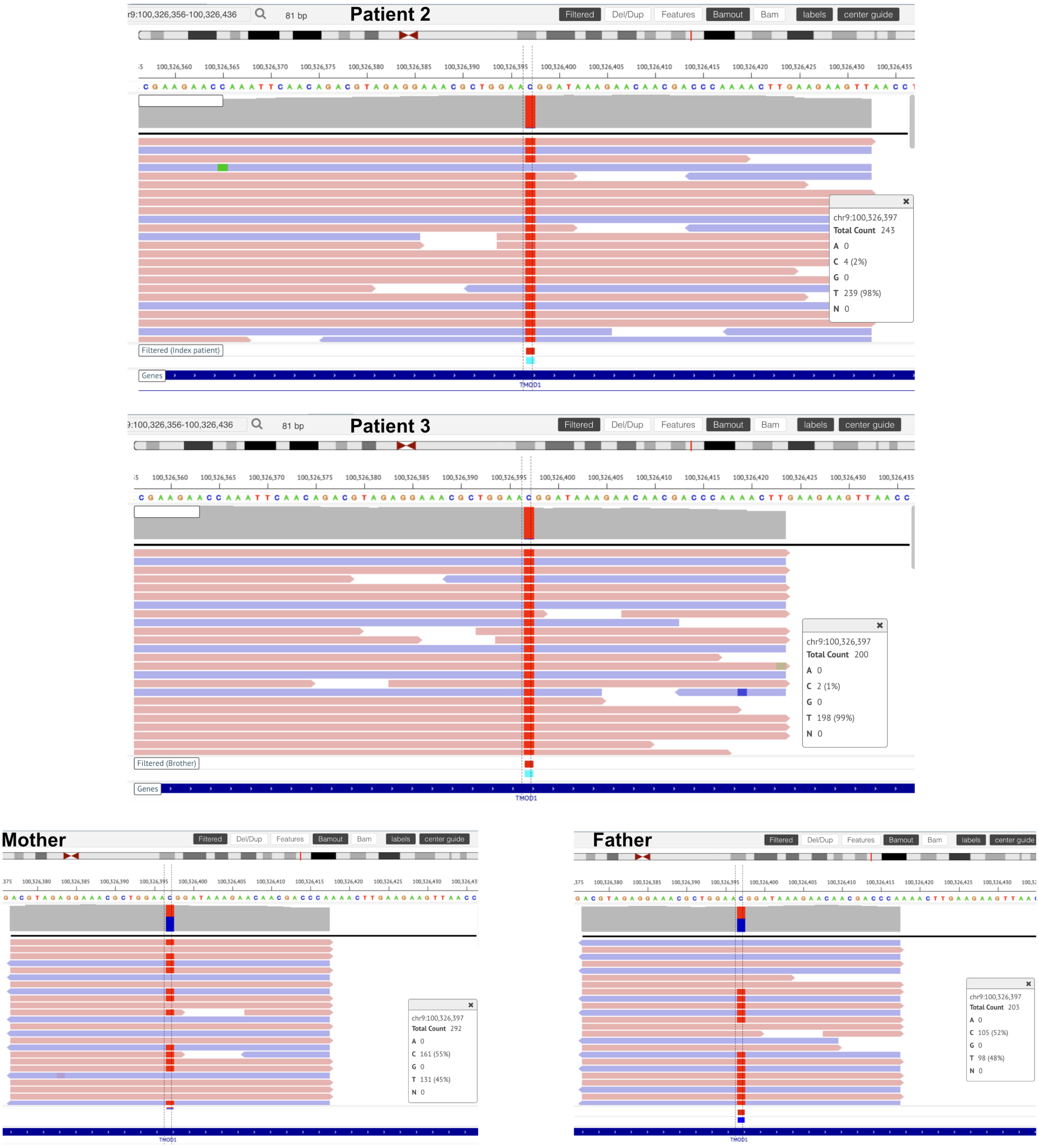

Supplementary Figure 3. Whole-exome sequencing data of Family 2. Integrated Genome Viewer visualization of WES data showing the TMOD1 variant in Family 2. Patients 2 and 3 are homozygous for TMOD1 variant c.565C>T, p.Arg189Trp. The parents are heterozygous carriers.

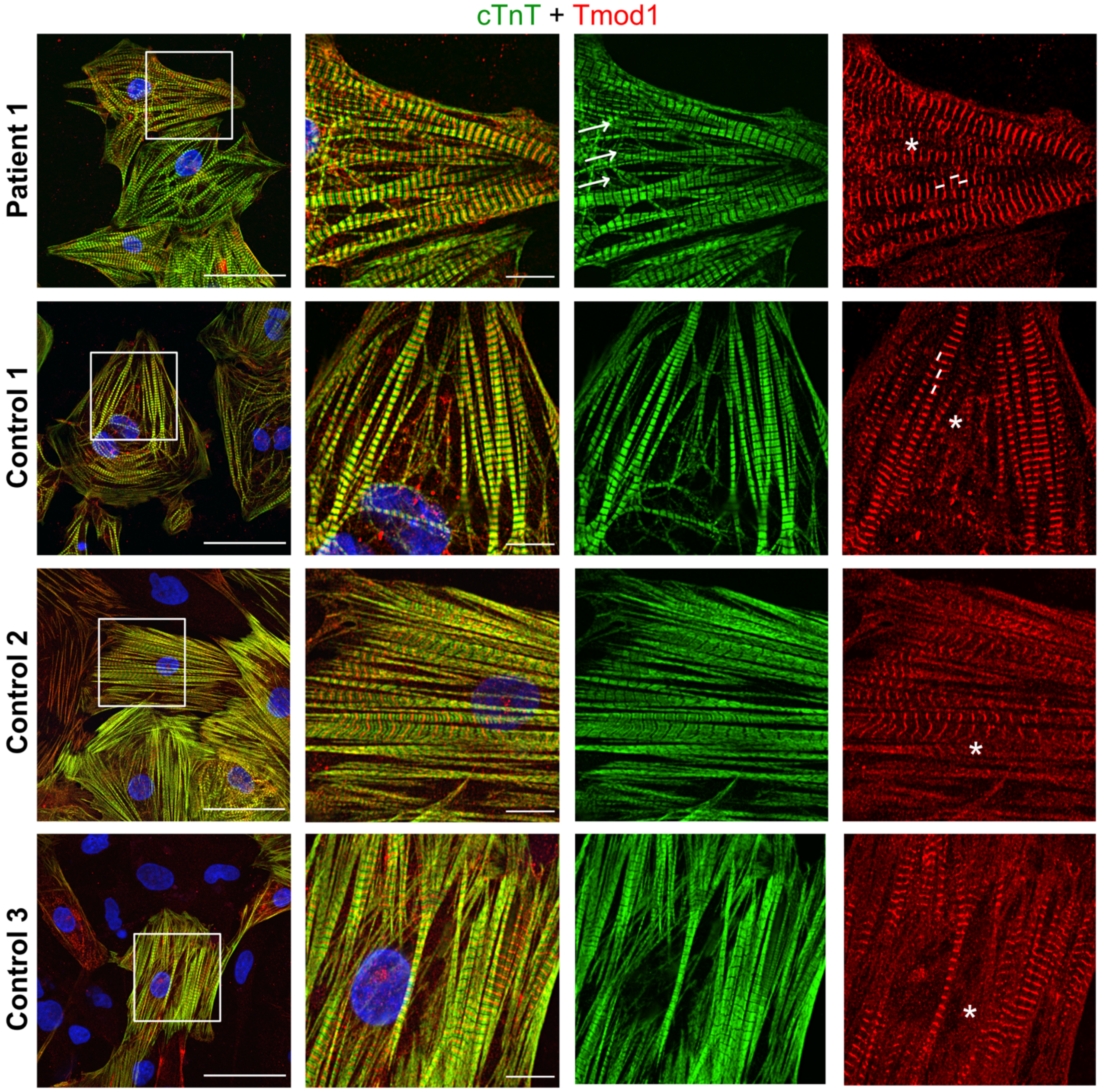

Supplementary Figure 4. Intracellular localization of TMOD1 does not disrupt the sarcomeric structure in iPSC-cardiomyocytes. TMOD1 localization in red in relation to cardiac troponin T (cTnT) in green in the Patient 1 and in control iPSC-cardiomyocytes. Scale bar = 50 μm and 10 μm. The white sarcomeric bars are drawn in controls at the same size as in the patient, to highlight approximately similar sarcomeric lengths. Asterisk: non-sarcomeric TMOD1.

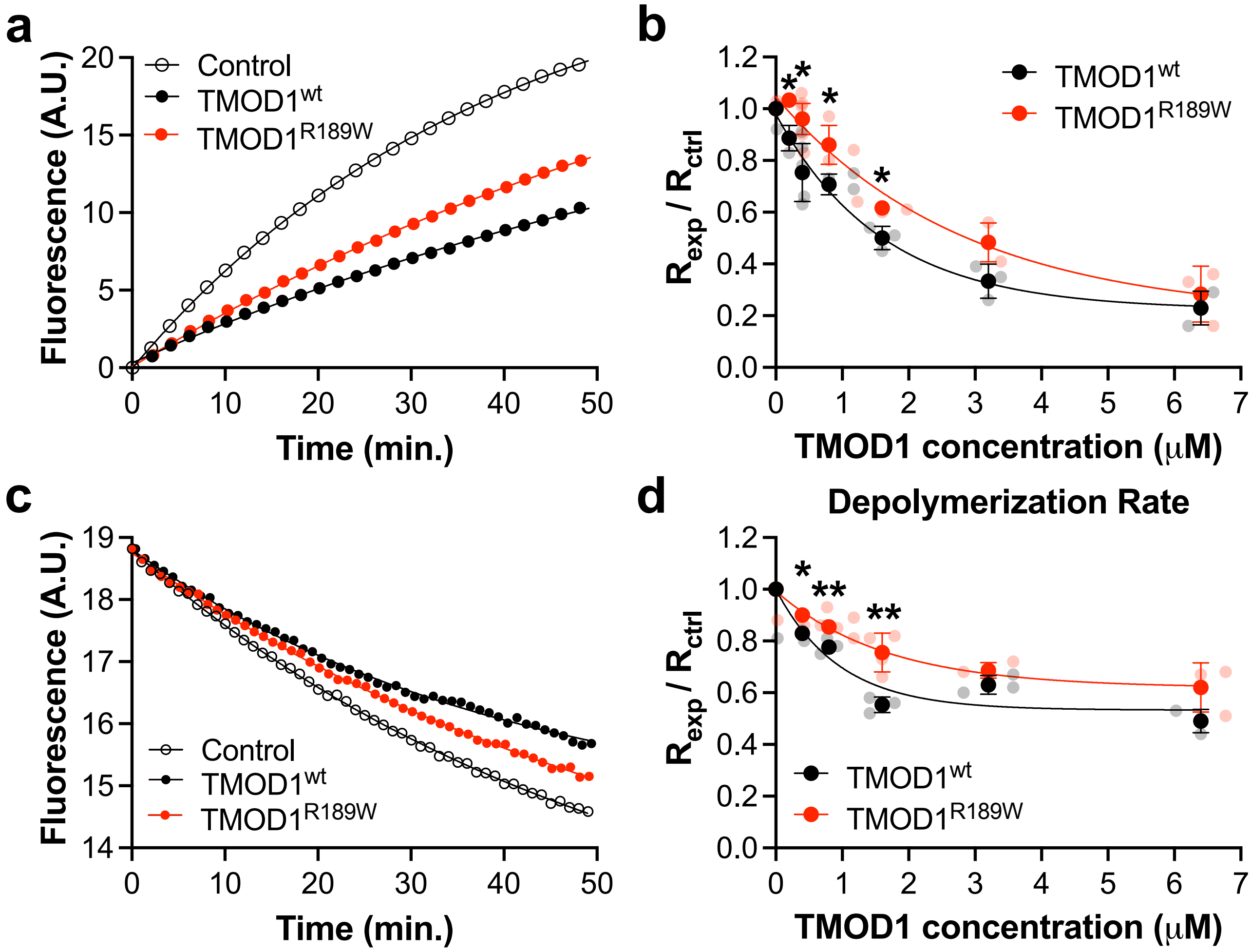

Supplementary Figure 5. The p.R189W variant weakens TMOD1's ability to cap the pointed ends of long actin filaments in the absence of TPM1. a Representative curves of pyrene-actin fluorescence (A.U.: arbitrary units) from pointed ends of gelsolin-capped actin filaments (1:100 gelsolin:actin molar ratio) over time (min) in the presence of 0.8 μM TMOD1^{wt} or TMOD1^{R189W} (control: spontaneous actin pointed end polymerization) **b** Concentration-dependent inhibition of actin polymerization in the presence of TMOD1^{wt} or TMOD1^{R189W} (*p*-values = 0.0101, 0.0483, 0.0118, 0.0495, 0.0607, 0.2809, respectively). **c** Representative curves of decrease in pyrene-actin fluorescence (A.U.) resulting from depolymerization from the pointed ends of gelsolin-capped actin filaments (1:100 gelsolin:actin molar ratio) over time (min) in the presence of 1.6 μM TMOD1^{wt} or TMOD1^{R189W} (control: spontaneous actin pointed end depolymerization) **d** Concentration-dependent inhibition of actin depolymerization in the presence of TMOD1^{wt} or TMOD1^{R189W} (*p*-values = 0.0152, 0.0087, 0.0076, 0.1064, 0.1005, respectively). Actin polymerization or depolymerization rates relative to the control (R_{exp} / $R_{control}$) were calculated as the first derivatives at time zero after exponential fit. Data shown are mean ± standard deviation (n=3 independent experiments, *$p<0.05$, **$p<0.01$, Student's t-test). Control, TMOD1^{wt} and TMOD1^{R189W} are shown in white, black and red circles, respectively.

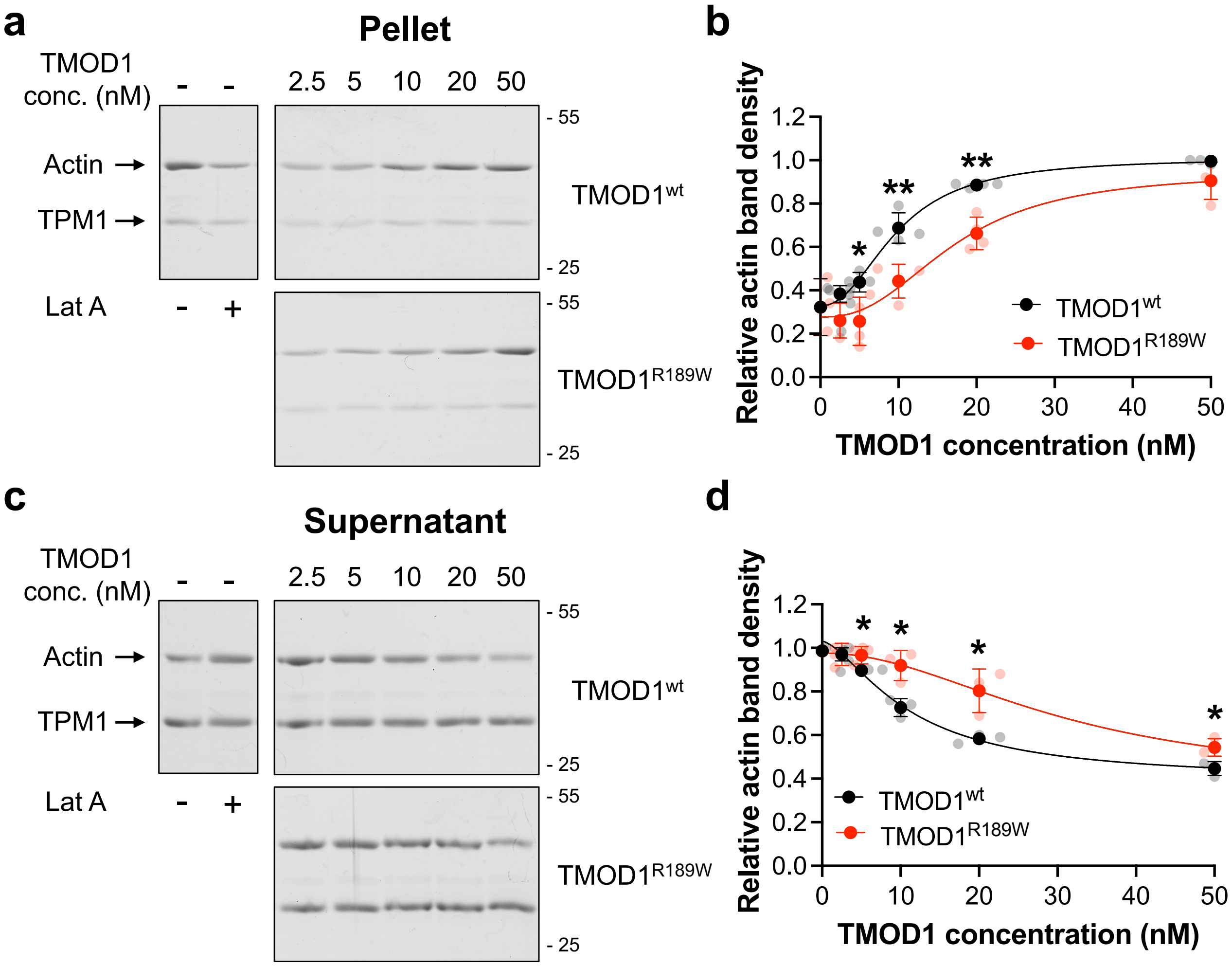

Supplementary Figure 6. The p.R189W variant weakens TMOD1's ability to inhibit Lat A-induced actin depolymerization from filament pointed ends. Coomassie-stained sodium dodecyl sulfate-polyacrylamide gels for **a** F-actin-containing pellet or **c** G-actin-containing supernatant samples resulting from ultracentrifugation of actin filaments after depolymerization by Lat A in the presence of tropomyosin (TPM1) and increasing concentrations of TMOD1. Concentration-dependent inhibition of actin depolymerization in the presence of $TMOD1^{wt}$ or $TMOD1^{R189W}$ was calculated from actin band densities from the **b** pellet (*p*-values = 0.0733, 0.0242, 0.0035, 0.0011, 0.0845, respectively) and **d** supernatant (*p*-values = >0.0999, 0.0412, 0.0147, 0.0204, 0.03016 respectively) samples quantified from sodium dodecyl sulfate-polyacrylamide gel electrophoresis. Data shown are mean ± standard deviation (n=3 or 4 independent experiments, $^{*}p<0.05$, $^{**}p<0.01$, Student's t-test). $TMOD1^{wt}$ and $TMOD1^{R189W}$ are shown in black and red circles, respectively.

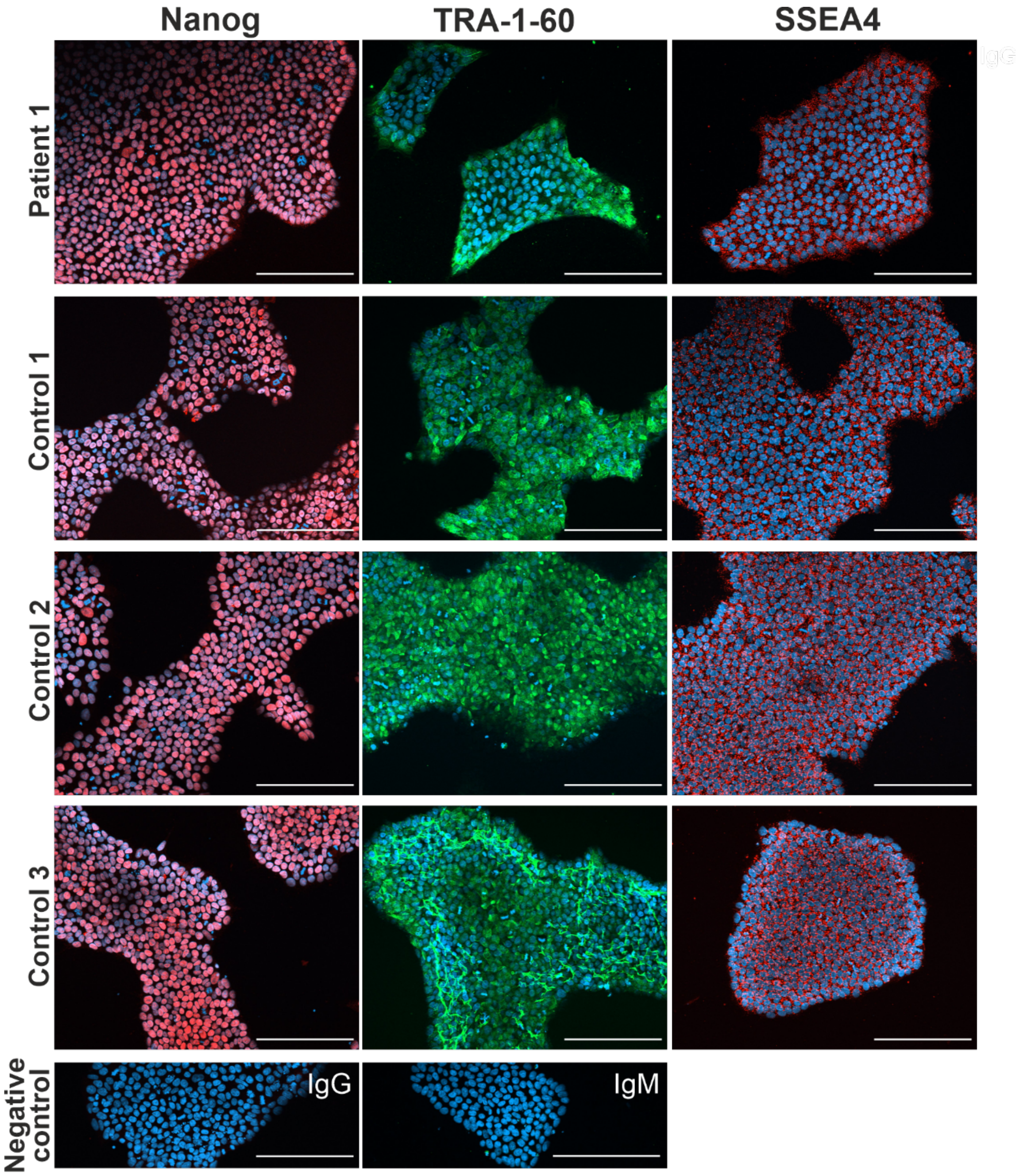

Supplementary Figure 7. Characterization of iPSCs by immunocytochemistry. iPSC lines derived from the fibroblasts of Patient 1 and three controls stained for the pluripotency markers Nanog (nuclear localization), TRA-1-60, and SSEA4 (stage-specific embryonic antigen 4) (surface markers). Scale bar = 500 µm.

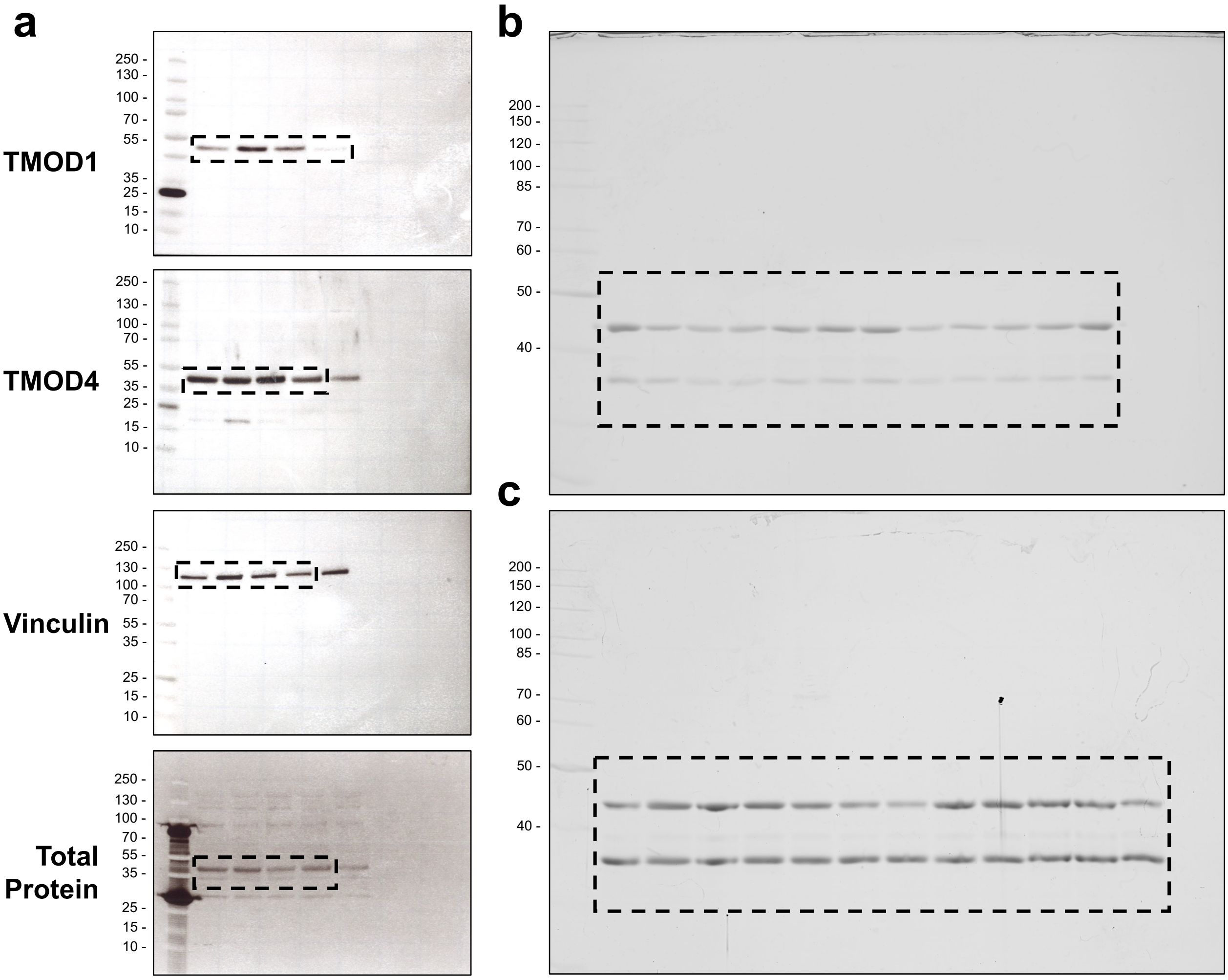

Supplementary Figure 8. Uncropped gels and blots for all figures. The areas used in **a** Fig. 2e, **b** Supplementary Fig. 6a, and **c** Supplementary Fig. 6c are marked by dashed boxes.
