# Supplementary material for: Recessive TMOD1 mutation causes childhood cardiomyopathy
Source: Commun Biol. 2024 Jan 2;7:7. doi: 10.1038/s42003-023-05670-9 (PMC10761686; doi:10.1038/s42003-023-05670-9)
Supplement: Supplementary file 3 — Description of Additional Supplementary Files [file 42003_2023_5670_MOESM3_ESM.pdf]

Description of Additional Supplementary Files

File name: Supplementary Data 1
Description: Source data for Figure 2 and Figure 4.

File name: Supplementary Video 1
Description: CMR imaging of Patient 1's heart.

File name: Supplementary Video 2
Description: CMR imaging of Patient 1's heart.

File name: Supplementary Video 3
Description: CMR imaging of Patient 2's heart.

File name: Supplementary Video 4
Description: CMR imaging of Patient 2's heart.

File name: Supplementary Video 5
Description: CMR imaging of Patient 3's heart.

File name: Supplementary Video 6
Description: CMR imaging of Patient 3's heart.
