# Supplementary material for: Recessive TMOD1 mutation causes childhood cardiomyopathy
Source: Commun Biol. 2024 Jan 2;7:7. doi: 10.1038/s42003-023-05670-9 (PMC10761686; doi:10.1038/s42003-023-05670-9)
Supplement: Supplementary file 11 — Reporting Summary [file 42003_2023_5670_MOESM11_ESM.pdf]

nature portfolio

Corresponding author(s): Anu Suomalainen, Carol C. Gregorio

Last updated by author(s): Nov 25, 2023

Reporting Summary

Nature Portfolio wishes to improve the reproducibility of the work that we publish. This form provides structure for consistency and transparency in reporting. For further information on Nature Portfolio policies, see our Editorial Policies and the Editorial Policy Checklist.

Statistics

For all statistical analyses, confirm that the following items are present in the figure legend, table legend, main text, or Methods section.

n/a	Confirmed	
☐	☒	The exact sample size (n) for each experimental group/condition, given as a discrete number and unit of measurement
☐	☒	A statement on whether measurements were taken from distinct samples or whether the same sample was measured repeatedly
☐	☒	The statistical test(s) used AND whether they are one- or two-sided<br>*Only common tests should be described solely by name; describe more complex techniques in the Methods section.*
☐	☒	A description of all covariates tested
☐	☒	A description of any assumptions or corrections, such as tests of normality and adjustment for multiple comparisons
☐	☒	A full description of the statistical parameters including central tendency (e.g. means) or other basic estimates (e.g. regression coefficient) AND variation (e.g. standard deviation) or associated estimates of uncertainty (e.g. confidence intervals)
☐	☒	For null hypothesis testing, the test statistic (e.g. F, t, r) with confidence intervals, effect sizes, degrees of freedom and P value noted<br>*Give P values as exact values whenever suitable.*
☒	☐	For Bayesian analysis, information on the choice of priors and Markov chain Monte Carlo settings
☒	☐	For hierarchical and complex designs, identification of the appropriate level for tests and full reporting of outcomes
☒	☐	Estimates of effect sizes (e.g. Cohen's d, Pearson's r), indicating how they were calculated

Our web collection on statistics for biologists contains articles on many of the points above.

Software and code

Policy information about availability of computer code

Data collection	Chemiluminescence images of immunoblots were captured with Image Lab Software (BioRad). ImageJ Version 1.52 (NIH) was used for collecting data from Coomassie-stained SDS-PAGE gels. ImageJ version 1.52 DDecon plug-in was used for thin filament length measurements. 3D deconvolution was performed using NIS offline deconvolution software (Nikon). Structural modeling of the TMOD1 homozygous variant was performed using Discovery Studio 4.5 (Biovia).
Data analysis	GraphPad Prism 9.0 was used for compiling the data, creating the figures and statistical analysis.

For manuscripts utilizing custom algorithms or software that are central to the research but not yet described in published literature, software must be made available to editors and reviewers. We strongly encourage code deposition in a community repository (e.g. GitHub). See the Nature Portfolio guidelines for submitting code & software for further information.

Data

Policy information about availability of data

All manuscripts must include a data availability statement. This statement should provide the following information, where applicable:

- Accession codes, unique identifiers, or web links for publicly available datasets
- A description of any restrictions on data availability
- For clinical datasets or third party data, please ensure that the statement adheres to our policy

The raw genetic data are not publicly available to preserve individuals' privacy under the European Data Protection Regulation. Data from main figures are available in Supplementary Data 1 and Supplementary Figure 8. All other data are available on request.

Research involving human participants, their data, or biological material

Policy information about studies with human participants or human data. See also policy information about sex, gender (identity/presentation), and sexual orientation and race, ethnicity and racism.

Reporting on sex and gender	The sex of human patients is provided in the manuscript.
Reporting on race, ethnicity, or other socially relevant groupings	Race or ethnicity of the patients are not provided to preserve the privacy of the patients under the European Data Protection Regulation.
Population characteristics	This manuscript did not study a population of subjects, only three patients from two unrelated families with the same homozygous gene mutation are reported.
Recruitment	The patients were recruited due to hospitalization from dilated and restrictive cardiomyopathy.
Ethics oversight	The study plan of the Childhood Cardiomyopathy project was approved by the Child and Adolescent Psychiatry Ethical Board and Coordinating Ethical Board of Helsinki University Hospital and received the ethical permit numbers 291/13/03/03/2008 and 254/13/03/00/14. All the samples were taken for diagnostic purposes with informed consent from parents and from patients when they were older than 10 years of age.

Note that full information on the approval of the study protocol must also be provided in the manuscript.

Field-specific reporting

Please select the one below that is the best fit for your research. If you are not sure, read the appropriate sections before making your selection.

☒ Life sciences ☐ Behavioural & social sciences ☐ Ecological, evolutionary & environmental sciences

For a reference copy of the document with all sections, see nature.com/documents/nr-reporting-summary-flat.pdf

Life sciences study design

All studies must disclose on these points even when the disclosure is negative.

Sample size	For the cell culture and recombinant protein studies, we initially aimed to perform 3 independent experiments in this manuscript. If data from a certain group(s) failed to reach to a sample size of 3 due to an inability of quantification resulting from technical difficulties (imperfections in gel/staining/culturing), the experiment was repeated for a fourth time in order to reach to a sample size of at least 3 for all groups and to achieve statistical significance.
Data exclusions	No data were excluded from analysis.
Replication	All attempts of replication were successful.
Randomization	Control cells (GFP-expressing or control iPSC-cardiomyocytes) were grouped and compared to the variant-expressing cells.
Blinding	For thin filament length measurements, folders containing data sets were blinded and revealed after analysis. For certain immunofluorescence sets, blinding was not possible since the treatment (i.e., presence of GFP fluorescence) had to be determined prior to collecting data.

Reporting for specific materials, systems and methods

We require information from authors about some types of materials, experimental systems and methods used in many studies. Here, indicate whether each material, system or method listed is relevant to your study. If you are not sure if a list item applies to your research, read the appropriate section before selecting a response.

Materials & experimental systems

n/a	Involved in the study
☐	☒ Antibodies
☐	☒ Eukaryotic cell lines
☒	☐ Palaeontology and archaeology
☐	☒ Animals and other organisms
☒	☐ Clinical data
☒	☐ Dual use research of concern
☒	☐ Plants

Methods

n/a	Involved in the study
☒	☐ ChIP-seq
☒	☐ Flow cytometry
☒	☐ MRI-based neuroimaging

Antibodies

Antibodies used	TNNT2 cardiac troponin T (1:300, Abcam, ab45932, host rabbit and 1:100, Novus Biologicals, MAB1874, host mouse)<br>TMOD1 Tropomodulin 1 (1:100, Novus Biologicals, NBP2-00955, host mouse)<br>SSEA4 (1:200, Thermo Fisher, MC-813-70, host mouse)<br>Tra-1-60 (1:50, Thermo Fisher, MC-813-70, host mouse)<br>Nanog (1:500, Cell Signaling Technologies,1E6C4, host mouse)<br>a-actinin (1:200, Sigma-Aldrich, EA-53, host mouse)
Validation	The tested antibodies were picked based on the manufacturers' recommendations on cross-reactivity and specificity. Known molecular weights of detected proteins were used as an indicator of specificity in immunoblots. For immunostaining of proteins, we compared our findings to published data to validate our results. Secondary antibodies alone were always used to determine the background signal.

Eukaryotic cell lines

Policy information about cell lines and Sex and Gender in Research

Cell line source(s)	Neonatal rat cardiomyocytes, mouse embryonic fibroblasts, iPSCs derived from human primary fibroblasts.
Authentication	None of the cell lines used were authenticated.
Mycoplasma contamination	Cells were not tested against mycoplasma contamination.
Commonly misidentified lines (See ICLAC register)	No misidentified cell lines were used.

Animals and other research organisms

Policy information about studies involving animals; ARRIVE guidelines recommended for reporting animal research, and Sex and Gender in Research

Laboratory animals	Neonatal mixed gender postnatal day 0-3 Sprague-Dawyley rats or mixed gender mouse embryos.
Wild animals	This study did not involve wild animals.
Reporting on sex	Hearts from mixed gender rats or mice were used for cell culture studies.
Field-collected samples	This study did not involve any field-collected samples.
Ethics oversight	The work with animals was performed under the approval by The Institutional Animal Care and Use Committee at the University of Arizona, Protocol number 08-017, which conformed to all applicable federal and institutional policies, procedures and regulations, including the PHS Policy on Humane Care and Use of Laboratory Animals, USDA regulations (9 CFR Parts 1, 2, 3), the Federal Animal Welfare Act (7 USC 2131 et. Seq.), the Guide for the Care and Use of Laboratory Animals, and all relevant institutional regulations and policies regarding animal care and use at the University of Arizona.

Note that full information on the approval of the study protocol must also be provided in the manuscript.
